# Trikafta—Extending Its Success to Less Common Mutations

**DOI:** 10.3390/jpm12091528

**Published:** 2022-09-19

**Authors:** Lea Bentur, Mordechai Pollak

**Affiliations:** 1 Pediatric Pulmonary Institute, Ruth Children’s Hospital, Rambam Health Care Center, Haifa 31096, Israel; 2 Faculty of Medicine, Technion-Israel Institute of Technology, Haifa 32000, Israel

Cystic Fibrosis (a genetic recessive disease) is caused by a mutant cystic fibrosis transmembrane conductance regulator (CFTR) gene that causes an absence of, or impaired CFTR activity. More than 2000 variants of CFTR gene have been reported, and they are grouped to six classes depending on the pathophysiology of the CFTR protein that functions as an anion channel transporter. Scientists’ understanding of this molecular biological defect has led to a revolutionary approach to mutation-specific therapy that can improve or even restore CFTR function. Various modulators and correctors have been developed based on the specific genotype and class of CFTR; hence, they represent a model of personalized medicine. The breakthrough in attitudes toward individualized therapy in CF has been further advanced by the development of in vitro models to predict responses to therapy; this has led to an innovative approach by the FDA of extending the approval of modulators to CF patients based solely on in vitro studies. The current study by Laselva et al. represents an excellent example of translational medicine bringing hope from bench to CF patients with orphan mutations.

In 2019, Trikafta was approved. This triple combination of two cystic fibrosis transmembrane conductance regulator (CFTR) correctors (Tezacaftor and Elexacaftor) and one CFTR potentiator (Ivakaftor) has been a significant game changer in the world of cystic fibrosis (CF). Trikafta has changed many people’s lives. In terms of lung function, in clinical trials, CF patients with one or two F508del mutations showed a markedly improved lung function gain of 10–14% with a reduction in exacerbations of 63% compared to those treated with Symdeko (the double combination of Tezacaftor and Ivacaftor) [1,2,3]. Lately Trikafta has also been found to be safe and efficient in the younger population aged 6–12 years [4]; consequently, Trikafta is now FDA-approved for all CF patients with at least one F508del mutation aged 6 and older. A striking finding that illustrates the incredible effect of Trikafta is the fact that most Trikafta-naïve patients listed for lung transplant could be safely unlisted after receiving Trikafta treatment [5]. Additional information about Trikafta and other CFTR-targeted medications can be found in the published reviews on this topic [6,7].

Although around 90% of CF patients carry at least one F508del mutation, and are therefore suitable for Trikafta therapy, the situation is more challenging for the others. Some of these patients, mainly those with non-sense mutations, cannot benefit from current CFTR modifiers and are still waiting for a scientific breakthrough [8]. However, hope exists for patients with rarer class 2 and class 3 CFTR mutations, since mutated CFTR shares characteristics with the more abundant mutations of the same class. In 2017 the FDA, in an innovative approach, approved Ivakaftor; this is a CFTR potentiator that was initially studied and approved for patients with the G551D mutation—and later, for patients with other mutations—based on the results of in vitro studies [9]. This demonstrated the concept that “Theratyping”—aligning genotypes based on their response to CFTR modulators—can be proven in vitro without large-scale randomized controlled trials that are not feasible for rare mutations [10]. Similarly, the FDA has approved Trikafta for patients baring mutations that exhibit an increase in the chloride transport of at least 10% of normal over baseline, based on in vitro data in Fischer Rat Thyroid (FRT) cells [11].

Laselva et al. [12] studied two rare CFTR mutations—H609R and I1023_V1024del—and the compound mutant I148T/I1023_V1024del. These mutations are known to cause a severe CF phenotype [13]. Using HEK-293 cells, they convincingly showed that these mutations cause reduced protein processing and altered channel function in vitro. They also showed that the functional defect is not further reduced in the compound mutant. Furthermore, they demonstrated that Trikafta, but not Orkambi (a combination of Lumakaftor and Ivakaftor), can partially prevent processing and functional defects in both mutations. This is in line with functional studies of H609R-CFTR in FRT cell line models that showed a residual function of CFTR with minimal response to Orkambi [14]. 

The authors recognize the limitations of their model, since it has been proven that protein processing can be dependent on the host cell type [15]. They correctly stress the need to corroborate their findings and probe the mechanism of drug action in relevant airway epithelial cells. 

With these limitations in mind, there is no doubt that this study gives hope to individuals who carry these specific mutations. Every effort should be made to gain access to tissue samples in order to perform in vitro studies that will meet the regulators’ requirements to approve Trikafta for these patients. Since the safety of Trikafta has been proven—and now, with considerable evidence that these patients could potentially benefit from the Trikafta therapy—it is also reasonable to perform an n-of-1 study in order to prove its efficacy in real life. 

Although the superiority of Trikafta over Symdeko and Orkambi is not doubted, this study can also give hope to patients who have had tissue samples tested in the past (intestinal organoids or nasal epithelial cells) and failed to show CFTR recovery with older CFTR modifiers. 

At last, studies such as that presented by Laselva et al. send a message of hope to the remaining CF patients who have not yet been approved for this game-changing medication. Researchers will not rest until all CF patients have access to highly potent medications such as Trikafta, no matter how rare their mutations are. 
